# Supplementary material for: Stringent and complex sequence constraints of an IGHV1-69 broadly neutralizing antibody to influenza Ha stem
Source: Cell Rep. Author manuscript; Available in PMC 2024 Feb 16. (PMC10872586; doi:10.1016/j.celrep.2023.113410)
Supplement: 1 [file NIHMS1948198-supplement-1.pdf]

Cell Reports, Volume *42*

Supplemental information

Stringent and complex sequence constraints of an IGHV1-69 broadly neutralizing antibody to influenza HA stem

Qi Wen Teo, Yiquan Wang, Huibin Lv, Timothy J.C. Tan, Ruipeng Lei, Kevin J. Mao, and Nicholas C. Wu

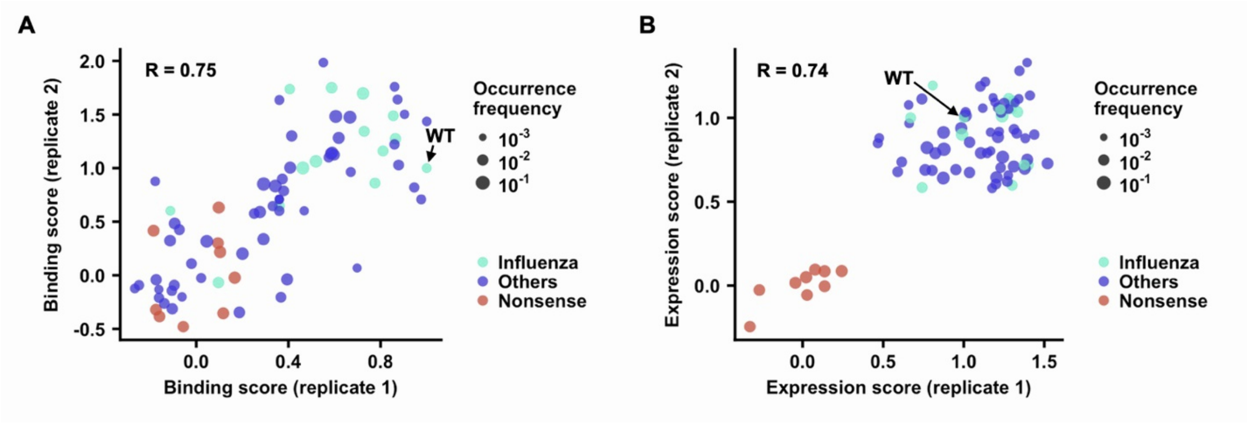

Figure S1. Correlation between biological replicates of binding and expression sorts of CR9114 light chain variant library, Related to Figure 1. (A) Correlation of binding scores between two independent biological replicates is shown. **(B)** Correlation of expression scores between two independent biological replicates is shown. The datapoint corresponding to CR9114 WT light chain is labeled as “WT”. Cyan: light chain variants from known IGHV1-69 antibodies to influenza virus. Blue: light chain variants from IGHV1-69 antibodies to non-influenza antigens. Red: negative control variants with premature stop codons. The Pearson correlation coefficient (R) is indicated.

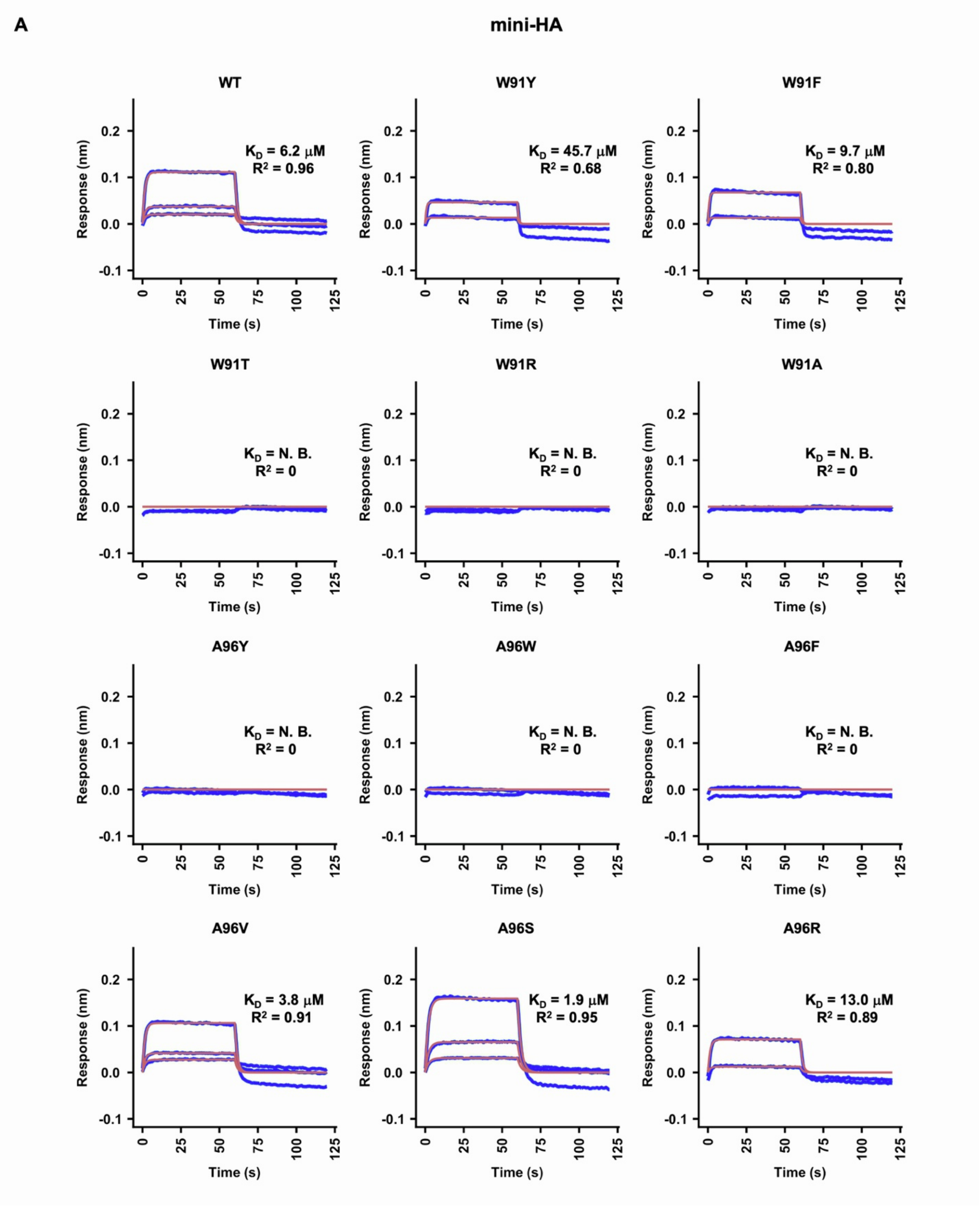
A
mini-HA
WT
K_D = 6.2 μM
R^2 = 0.96
W91Y
K_D = 45.7 μM
R^2 = 0.68
W91F
K_D = 9.7 μM
R^2 = 0.80
W91T
K_D = N. B.
R^2 = 0
W91R
K_D = N. B.
R^2 = 0
W91A
K_D = N. B.
R^2 = 0
A96Y
K_D = N. B.
R^2 = 0
A96W
K_D = N. B.
R^2 = 0
A96F
K_D = N. B.
R^2 = 0
A96V
K_D = 3.8 μM
R^2 = 0.91
A96S
K_D = 1.9 μM
R^2 = 0.95
A96R
K_D = 13.0 μM
R^2 = 0.89
Response (nm)
Time (s)

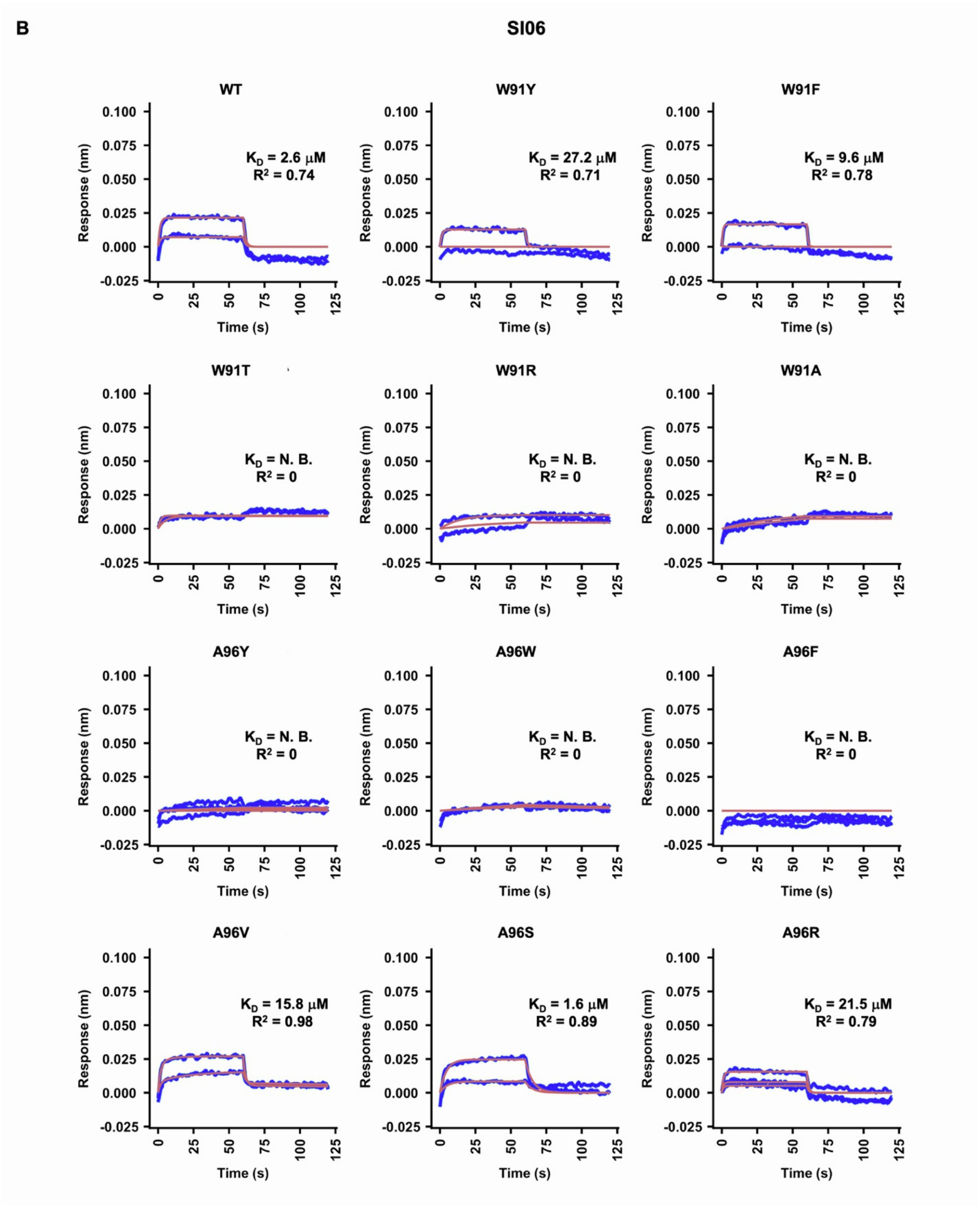

Figure S2. Sensorgrams for binding of CR9114 gHC mutants to mini-HA and SI06 HA, Related to Figure 2 and Table 1. Binding kinetics of different Fabs against **(A)** mini-HA and **(B)**

SI06 were measured by biolayer interferometry (BLI). Y-axis represents the response. Blue lines represent the response curve and red lines represent the best fit model (1:1 binding model or 2:1 heterogeneous ligand model, see STAR Methods). Binding kinetics were measured for two to three Fab concentrations (33 nM, 100 nM and 300 nM). Dissociation constant (K_D) and the goodness of model fitting (R^2) are indicated. N.B. indicates no binding.

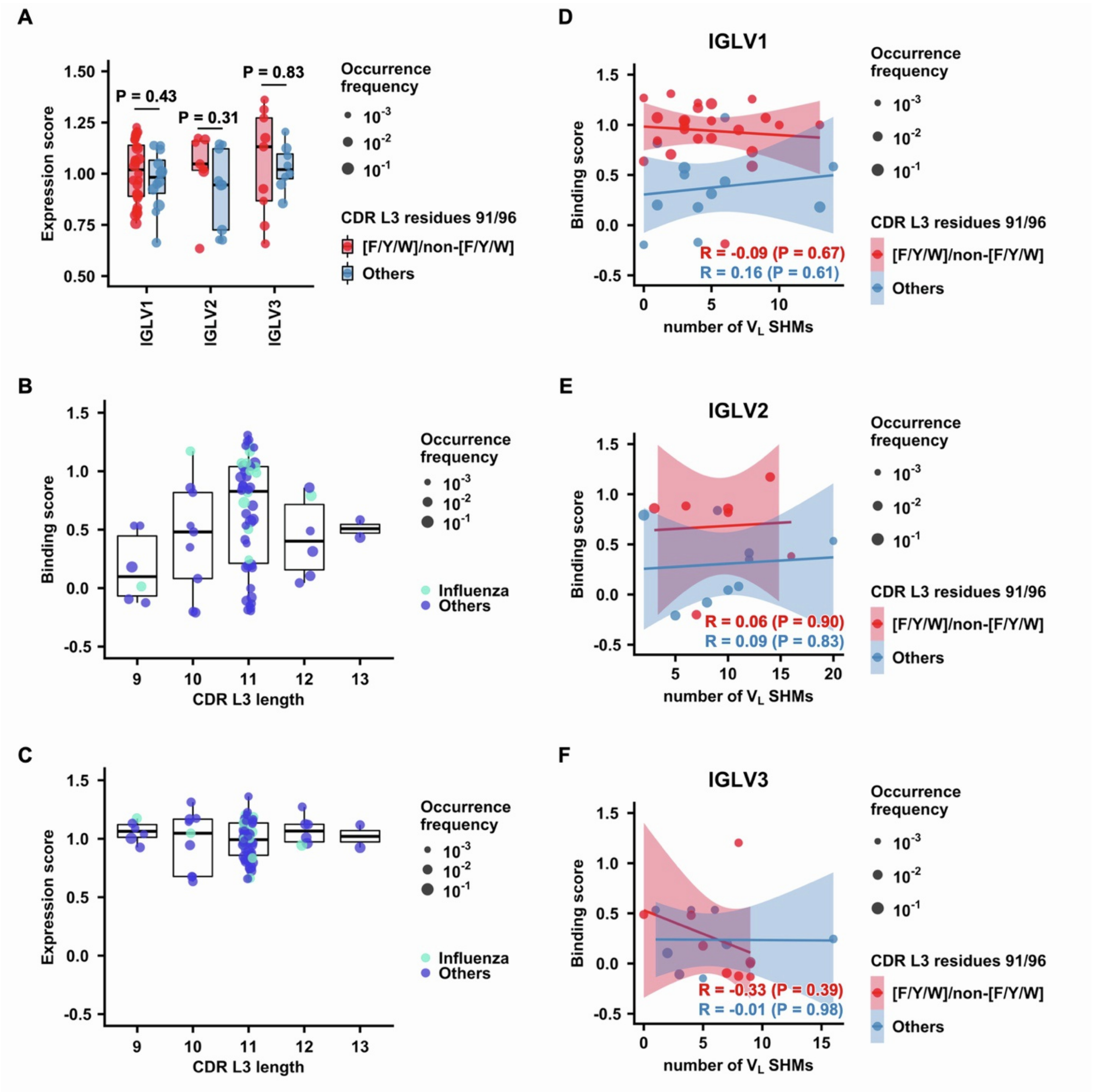

Figure S3. Impact of CDR L3 length and somatic hypermutation (SHM) on HA stem binding activity of CR9114, Related to Figure 2. (A) Expression scores of light chain variants in different IGLV families with and without V_L $91_{F/Y/W}/96_{non\text{-}F/Y/W}$ are compared. Red: with V_L $91_{F/Y/W}/96_{non\text{-}F/Y/W}$; Blue: without V_L $91_{F/Y/W}/96_{non\text{-}F/Y/W}$. P-values were computed by two-tailed Student's t-test. **(B-C)** Binding **(B)** and expression **(C)** scores of light chain variants with different CDR L3 lengths are compared. Cyan: light chain variants from known IGHV1-69 antibodies to influenza virus. Blue:

light chain variants from IGHV1-69 antibodies to non-influenza antigens. **(D-F)** Correlation between binding score and the number V_L SHM is shown for light chain variants in different light chain families, namely IGLV1 **(D)**, IGLV2 **(E)**, and IGLV3 **(F)**. The Pearson correlation coefficient (R) is indicated. P-values were computed by Pearson correlation test.

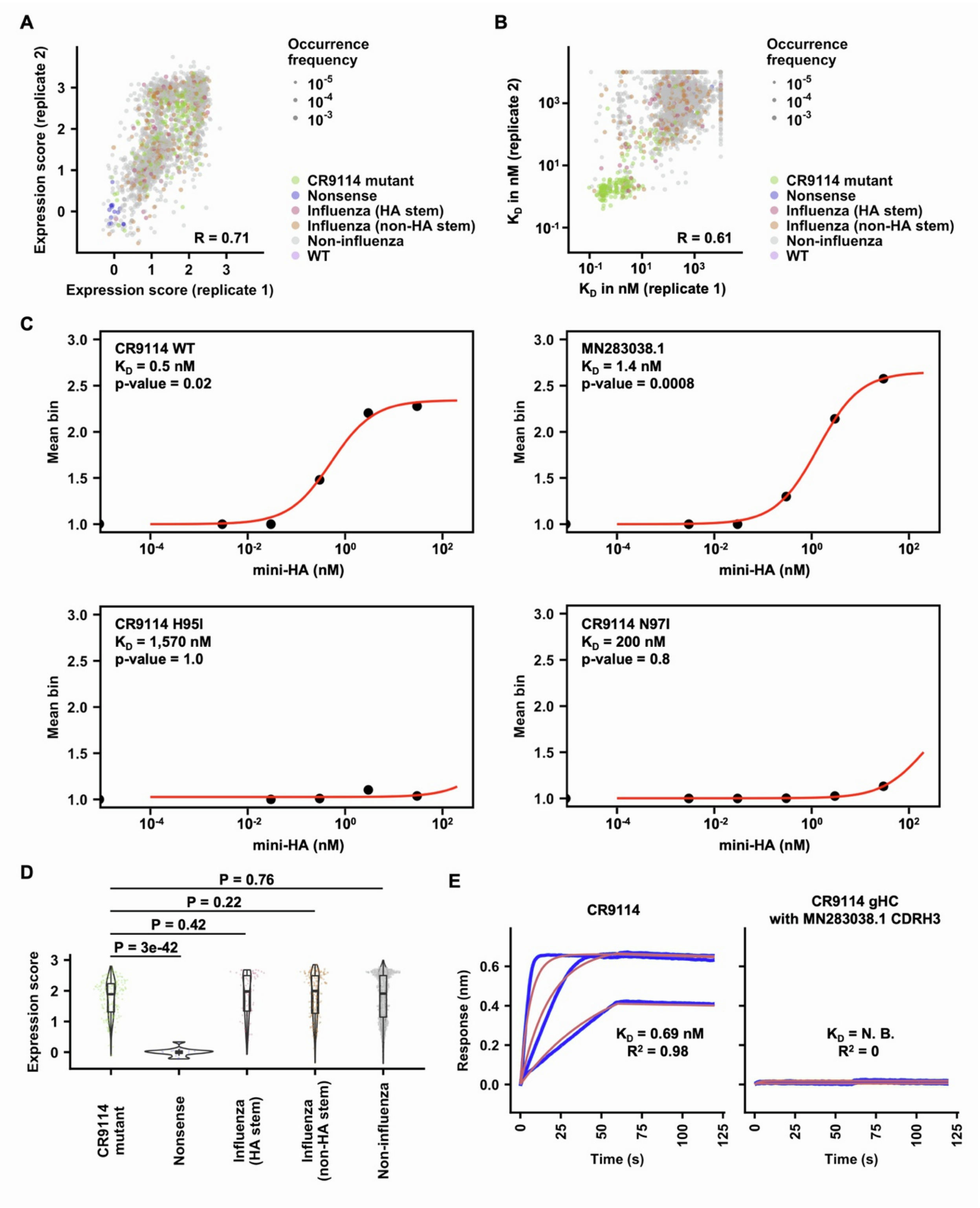

Figure S4. Reproducibility and analysis of Tite-Seq for CDR H3 variants, Related to Figure 3. (A) Correlation of expression scores between two independent biological replicates is shown.

(B) Correlation of apparent dissociation constant (K_D) values between two independent biological replicates is shown. **(C)** Example titration curves inferred from Tite-Seq data. **(D)** Distributions of expression scores for CDR H3 variants from different types of antibodies. Greenish yellow: CR9114 single amino acid mutants; purple: nonsense variants with stop codons; red: CDR H3 variants from IGHV1-69 HA stem antibodies; orange: CDR H3 variants from IGHV1-69 non-HA stem influenza antibodies; grey: CDR H3 variants from IGHV1-69 non-influenza antibodies; pink: WT. **(E)** Binding kinetics of a CDR H3 variant from an IGHV1-69 non-influenza antibody (Genbank ID: MN283038.1) and CR9114 (positive control) to mini-HA were measured by BLI. Y-axis represents the response. Blue lines represent the response curve and red lines represent a 1:1 binding model. Binding kinetics were measured for at least two Fab concentrations. N.B. indicates no binding. Dissociation constant (K_D) and the goodness of model fitting (R^2) are indicated.

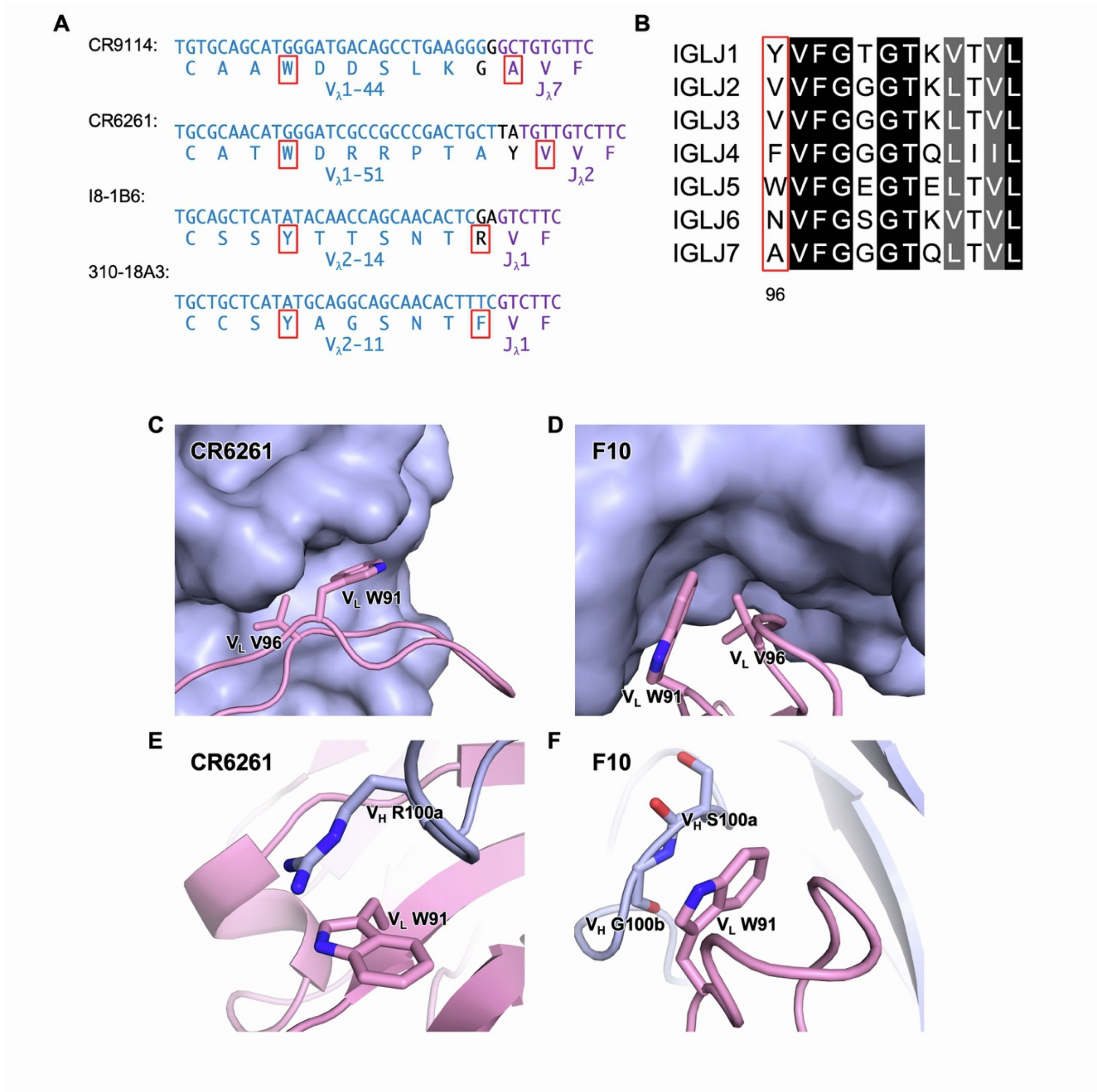

Figure S5. Additional characterizations of IGHV1-69 HA stem antibodies, Related to Figure 4. (A) Nucleotide and amino acid sequences of light chain V-J junction are shown for different IGHV1-69 HA stem antibodies. V_L residues 91 and 96 are indicated in red. Blue: V-region; purple: J-region; black: N-region. **(B)** CDR L3 sequences among different IGLJ families. V_L residue 96 is indicated in red. **(C-D)** Side chains of V_L W91 and A96 at the heavy-light chain interfaces of **(C)** CR6261 and **(D)** F10 are shown as sticks representation. Heavy chain is in light blue surface representation. Light chain is colored in pink. **(E)** π-π stacking interaction between V_H R100a and V_L W91 of CR6261 is shown. **(F)** π-π stacking interaction between V_H S100a-G100b peptide bond

and V_L W91 of F10 is shown. PDB 3GBN [S1] and PDB 3FKU [S2] are used for CR6261 and F10, respectively. V_H and V_L indicate variable regions of antibody heavy and light chains, respectively.

Table S4. List of primers used in this study, Related to STAR Methods.

Primer Name	Sequence (5′ to 3′)
V_H1-69-Lightchain-lib-VF	GGACAACCAAAGGCTGCTCCTTC
V_H1-69-LightChain-lib-VR	GGCCGGCTGGGCCGCTGCTAAAACTGA
V_H1-69-LightChain-lib-IF	TTTCAATATTTTCTGTTATTGCTTCAGTTTTAGCAGCGGCCCAGCCGGCC
V_H1-69-LightChain-lib-IR	TCAGAGGATGGAGGGAACAAGGTGACAGAAGGAGCAGCCTTTGGTTGTCC
V_H1-69-LightChain-recover-F	CAGTTTTAGCAGCGGCCCAGCCG
V_H1-69-LightChain-recover-R	ACAGAAGGAGCAGCCTTTGGTTG
V_H1-69-CDRH3-VF	GGCCAAGGGACCACGGTCACCGTCTCCTCAGCTTC
V_H1-69-CDRH3-VR	GTAATACACGGCCGTGTCCTCAGATCTCAGGCTGC
V_H1-69-CDRH3-lib-F	CACAGCCTACATGGAGCTGAGCAGCCTGAGATCTGAGGACACGGCCGTGTATTAC
V_H1-69-CDRH3-lib-R	AAAACGGAAGGTCCCTTAGTAGAAGCTGAGGAGACGGTGACCGTGGTCCCTTGGC
V_H1-69-CDRH3-recover-F	ATCTGAGGACACGGCCGTGTATTAC
V_H1-69-CDRH3-recover-R	AGACGGTGACCGTGGTCCCTTGGCC

Supplemental References

S1. Ekiert, D.C., Bhabha, G., Elsliger, M.A., Friesen, R.H., Jongeneelen, M., Throsby, M., Goudsmit, J., and Wilson, I.A. (2009). Antibody recognition of a highly conserved influenza virus epitope. Science *324*, 246-251. 10.1126/science.1171491.

S2. Sui, J., Hwang, W.C., Perez, S., Wei, G., Aird, D., Chen, L.M., Santelli, E., Stec, B., Cadwell, G., Ali, M., et al. (2009). Structural and functional bases for broad-spectrum neutralization of avian and human influenza A viruses. Nat Struct Mol Biol *16*, 265-273. 10.1038/nsmb.1566.
